# Cardioprotective effects of tanshinone IIA pretreatment via kinin B2 receptor-Akt-GSK-3β dependent pathway in experimental diabetic cardiomyopathy

**DOI:** 10.1186/1475-2840-10-4

**Published:** 2011-01-13

**Authors:** Dongdong Sun, Min Shen, Jiayi Li, Weijie Li, Yingmei Zhang, Li Zhao, Zheng Zhang, Yuan Yuan, Haichang Wang, Feng Cao

**Affiliations:** 1 This work was performed in Xijing Hospital, Fourth Military Medical University, Xi'an, Shaanxi, 710032, China

## Abstract

**Aims:**

Diabetic cardiomyopathy, characterized by myocardial structural and functional changes, is a specific cardiomyopathy develops in patients with diabetes mellitus. The present study was to investigate the role of kinin B2 receptor-Akt-glycogen synthase kinase (GSK)-3β signalling pathway in mediating the protective effects of tanshinone IIA (TSN) on diabetic cardiomyopathy.

**Methods and results:**

Streptozocin (STZ) induced diabetic rats (n = 60) were randomized to receive TSN, TSN plus HOE140 (a kinin B2 receptor antagonist), or saline. Healthy Sprague-Dawley (SD) rats (n = 20) were used as control. Left ventricular function, myocardial apoptosis, myocardial ultrastructure, Akt, GSK-3β and NF-κB phosphorylation, the expression of TNF-α, IL-6 and myeloperoxidase (MPO) were examined. Cardiac function was well preserved as evidenced by increased left ventricular ejection fraction (LVEF) and ± dp/dt (maximum speed of contraction/relaxation), along with decreased myocardial apoptotic death after TSN administration. TSN pretreatment alleviated mitochondria ultrastructure changes. TSN also enhanced Akt and GSK-3β phosphorylation and inhibited NF-κB phosphorylation, resulting in decreased TNF-α, IL-6 and MPO activities. Moreover, pretreatment with HOE140 abolished the beneficial effects of TSN: a decrease in LVEF and ± dp/dt, an inhibition of cardiomyocyte apoptosis, a destruction of cardiomyocyte mitochondria cristae, a reduction of Akt and GSK-3β phosphorylation, an enhancement of NF-κB phosphorylation and an increase of TNF-α, IL-6 and MPO production.

**Conclusion:**

These data indicated that TSN is cardioprotective in the context of diabetic cardiomyopathy through kinin B2 receptor-Akt-GSK-3β dependent pathway.

## Introduction

Diabetic cardiomyopathy is characterized by cardiac dysfunction with subsequent heart failure in patients with diabetes mellitus in the absence of coronary atherosclerosis. Over the last decades, there are numerous studies investigating the underlying pathological mechanisms of diabetic cardiomyopathy using animal models of diabetes mellitus as well as clinical data from diabetic patients. There is evidence that changes in the extracellular matrix with increased cardiac fibrosis [1-3], excessive generation of reactive oxygen species [4], as well as cardiac inflammation [5-7], characterized by increased levels of pro-inflammatory cytokines may play a role in the manifestation of diabetic cardiomyopathy.

The kallikrein-kinin system (KKS), which was shown to exist in the cardiac tissue as a local system [8], might have beneficial effects in diabetic cardiomyopathy [6]. The effects of kinins are mediated by stimulation of specific receptors, classified as kinin B1 receptor (B1R) and kinin B2 receptor (B2R). To date, more knowledge exists about the function of the B2R, which is thought to mediate most of the known cardiovascular beneficial effects of kinins.

Tanshinone ⅡA (TSN), one of the most abundant components of tanshinones, exhibits a variety of cardiovascular activities including vasorelaxation and protection against ischemia-reperfusion injury and antiarrhythmic effects [9-11]. The safety of TSN treatment has been well established after its widespread application in the treatment of angina pectoris, acute ischemic stroke and arrhythmia in Asian countries. However, the effects and mechanisms of TSN on experimental diabetic cardiomyopathy are not well understood. Therefore, the aims of the present study were 1) to determine whether TSN protects against experimental diabetic cardiomyopathy; 2) to identify the role of B2R in the mechanism responsible for the effects of TSN.

## Methods

### Animals procedures

HOE140 was used as a kinin B2 receptor antagonist. Eighty Sprague-Dawley (SD) rats, weight 200 to 220 g, were randomly allocated into the following groups with n = 20 each: (1) DM; (2) DM + TSN (TSN); (3) DM + TSN + HOE140 + I/R (HOE140); (4) control. Diabetes mellitus (DM) was induced in group (1), (2) and (3) by intraperitoneal injections of streptozocin (STZ) (50 mg/kg, STZ was dissolved in 0.1 M citrate buffer, pH 4.5) as previously described [12].

A one-drop blood sample was obtained at 1, 7 and 12 weeks after STZ injection from all rats through the tip of the tail for the determination of blood glucose concentration by using a reflectance meter (Accu-Chek, Roche Diagnostics GmbH, Mannheim, Germany). Eleven weeks after STZ was given, TSN (5 mg/kg) was administered by i.p. injection for seven days. HOE140 (10 μg/kg) was injected via the tail vein 10 min before TSN injection. Control group received the same volume of 0.9% saline by i.p. injection for seven days.

**Figure 1:**
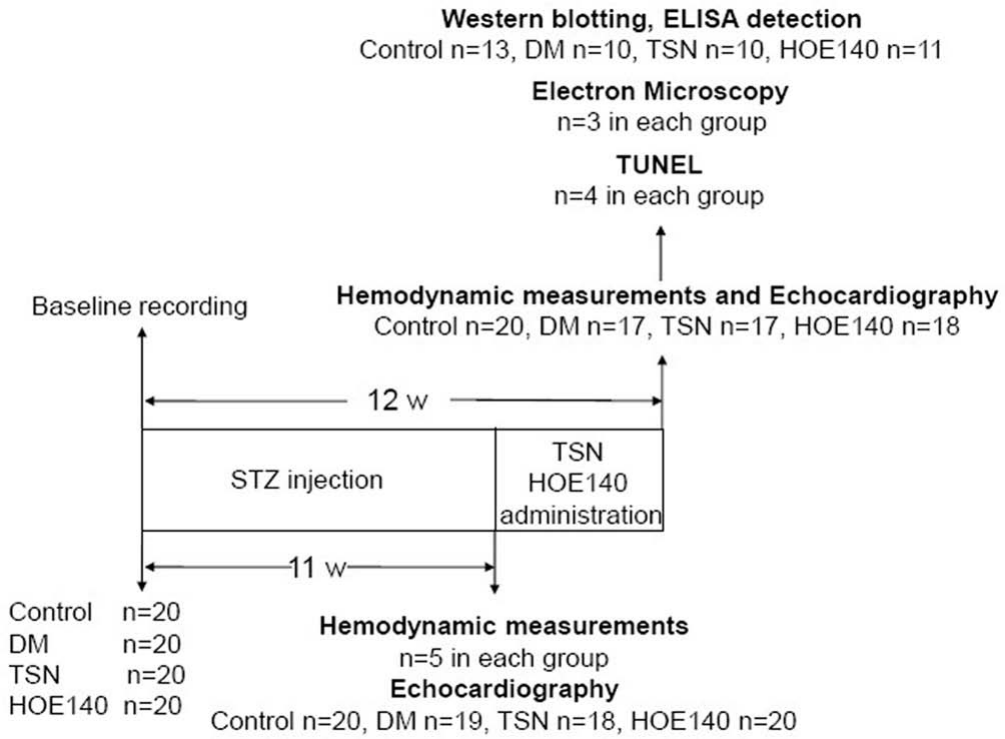
Study design

One day before, 11 weeks and 12 weeks after STZ was given, Invasive cardiac hemodynamic analysis was performed similar as previous described [13,14]. In brief, rats were anesthetized with 3% isoflurane. The left ventricular pressure (LVP) was measured via a Millar Mikro-tip catheter transducer that was inserted into the left ventricular cavity through the left carotid artery. The left ventricular systolic pressure (LVSP), left ventricular end-diastolic pressure (LVEDP), first derivative of the left ventricular pressure (+dp/dt max and -dp/dt max) and heart rate were obtained by use of computer algorithms and an interactive videographics program (Po-Ne-Mah Physiology Platform P3 Plus, Gould Instrument Systems, Valley View, Ohio). Of 80 rats, 20 in control group, 17 in DM group, 17 in TSN group, 18 in HOE140 group survived the surgery and saline/STZ/TSN/HOE140 administration (Figure 1). The experiments were performed in adherence with the National Institutes of Health Guidelines on the Use of Laboratory Animals and were approved by the Fourth Military Medical University Committee on Animal Care.

### Determination of cardiac function

Cardiac function and dimensions were assessed at 1 day before, 11 weeks and 12 weeks after STZ was given using an echocardiography system (Sequoia Acuson, Siemens; 15-MHz linear transducer) under 3% isoflurane inhalation via a nose cone. Cardiac dimensions and function were assessed by M-mode echocardiography. Left ventricular end-diastolic diameter (LVEDD) and Left ventricular end-systolic diameter (LVESD) were measured on the parasternal left ventricular long axis view, All measurements represent the mean of 5 consecutive cardiac cycles. Left ventricular end-systolic volume (LVESV), Left ventricular end-diastolic volume (LVEDV) and Left ventricular ejection fraction (LVEF) were calculated by use of computer algorithms. All of these measurements were performed in a blinded manner.

### Electron microscopy

After echocardiography assessment, rats were anesthetizing with 3% sodium pentobarbital, hearts were rapidly removed and washed with PBS solution. At a low temperature, a specimen of the left ventricular myocardium removed with ophthalmic scissors was cut into a 1 mm tissue mass. Images were taken after fixation, soaking, stepwise alcohol dehydration, displacement, embedding, polymerization, sectioning, and staining and observed with an electron microscope (JEM-2000EX TEM, Japan). Random sections were taken and analysed by two technicians blinded to the treatments.

### Determination of myocardial apoptosis

Myocardial apoptosis was determined by terminal deoxyribonucleotidyl transferase-mediated dUTP-biotin nick end labeling (TUNEL) staining and caspase 3 activity assay, as described previously [13]. Apoptotic index (AI) = number of TUNEL-positive myocytes/total number of myocytes stained with DAPI from a total of 40 fields per heart (n = 4). Caspase-3 activity was measured with the ApoAlert Caspase-3 Assay Plate (Clontech, Mountain View, Calif) according to the manufacturer's instructions. All of these assays were performed in a blinded manner.

### Western blot evaluation and ELISA detection of cytokine levels

Protein was isolated from homogenized heart tissue with Trizol reagent (Invitrogen, Carlsbad, Calif) and standard Invitrogen protocols as previously described [12,13]. The Bradford assay (Bio-Rad Laboratories, Hercules, Calif) was used to quantify protein concentrations. Protein was then used for Western blotting with primary antibodies against Akt, p-Akt (ser 473), p-GSK-3β (ser 9), GSK-3β, p-NF-κB p65 (ser 536). All of the antibodies were purchased from Santa Cruz Biotechnology (Santa Cruz, Calif). The blots were visualized with a chemiluminescene system (Amersham Bioscience, Buchinghamshire, UK). The signals were quantified by densitometry. Concentrations of TNF-α and IL-6 were measured by commercially available enzyme-linked immunosorbent assay (ELISA) kits according to the manufacture's instructions. Values are expressed as pg/mg of total protein.

### Statistical analysis

Continuous variables that approximated the normal distribution were expressed as means ± SD. Comparison between groups were subjected to ANOVA followed by Bonferroni correction for post hoc t test. Data expressed as proportions were assessed with a Chi-square test or Fisher's exact test, as appropriate. Two sided tests have been used throughout, and *P *values < 0.05 were considered statistically significant. SPSS software package version 14.0 (SPSS, Chicago, IL) was used for data analysis.

## Results

### Basic parameters

Heart rate had no statistical differences between groups both at baseline and at 12 weeks after diabetes was induced. Blood glucose was significantly elevated in STZ injection groups (DM, TSN, HOE140) compared with baseline. Compared with TSN group, diabetic rats in group DM and group HOE140 had significantly smaller body weight 12 weeks after diabetes was induced. Heart to body mass ratio was significantly higher in DM and HOE140 group compared with TSN group 12 weeks after STZ injection (Table 1).

**Table 1:** Basic parameters of diabetic rats

Basic Parameters	Control (n = 20)	DM (n = 17)	TSN (n = 17)	HOE140 (n = 18)
Heart rate (min^-1^)				
baseline	425.2 ± 12.1	427.6 ± 14.6	420.6 ± 12.1	421.3 ± 15.1
12 w	421.4 ± 29.1	418.1 ± 12.9	415.7 ± 11.6	415.8 ± 14.2
Blood glucose (mmol/L)				
baseline	5.1 ± 0.4	5.1 ± 0.5	5.2 ± 0.5	5.1 ± 0.5
12 w	5.1 ± 0.5	25.1 ± 1.1*	24.4 ± 1.1*	25.6 ± 2.7*
Body mass (g)				
baseline	197.9 ± 12.5	205.2 ± 12.3	204.1 ± 12.3	206.1 ± 13.2
12 w	531.7 ± 14.9	418.0 ± 14.9*	441.4 ± 20.2*^#^	412.8 ± 12.3*^##^
Heart to body mass ratio (mg/g)				
baseline	1.94 ± 0.19	1.96 ± 0.25	1.90 ± 0.23	1.94 ± 0.26
12 w	1.95 ± 0.21	2.55 ± 0.33*	2.34 ± 0.30*^#^	2.46 ± 0.29*

Values are presented as mean ± SD.

*p < 0.05 *vs *Control, ^# ^*p *< 0.05 *vs *DM, ^## ^*p *< 0.05 *vs *TSN.

### TSN preserves left ventricular function in diabetic rats

Hemodynamic measurements were performed at baseline, 11 w and 12 w after STZ injection (Table 2). In diabetic hearts, maximum speed of contraction (+ LV dp/dt max) was significantly decreased as compared with the control group (3588.1 ± 454.0 *vs *4502.4 ± 254.1 mmHg/s, *P *< 0.05). TSN administration enhanced + LV dp/dt max as compared with the diabetes group (4180.0 ± 747.9 *vs *3588.1 ± 454.0 mmHg/s, *P *< 0.05). TSN pretreatment also significantly increased the maximum speed of relaxation (- LV dp/dt max) (3878.4 ± 526.2 *vs *3389.9 ± 380.9 mmHg/s, *P *< 0.05) as compared with the diabetes group. TSN's effects on + LV dp/dt max (4180.0 ± 747.9 mmHg/s in TSN group *vs *3627.3 ± 199.0 mmHg/s in HOE140 group, *P *< 0.05) and - LV dp/dt max (3878.4 ± 526.2 mmHg/s in TSN group *vs *3335.7 ± 499.0 mmHg/s in HOE140 group, *P *< 0.05) were abolished by HOE140.

**Table 2:** Left Ventricular Function Evaluation by Hemodynamic Measurements and Echocardiograpy

Parameters	Control (n = 20)	DM (n = 17)	TSN (n = 17)	HOE140 (n = 18)
+LV dp/dt max				
baseline	4449.8 ± 190.2	4458.9 ± 318.7	4462.5 ± 253.5	4430.9 ± 455.8
11 w	4528.2 ± 220.4	3678.6 ± 335.2*	3759.8 ± 372.1*	3699.3 ± 257.2*
12 w	4502.4 ± 254.1	3588.1 ± 454.0*	4180.0 ± 747.9^#^	3627.3 ± 199.0* ^##^
-LV dp/dt max				
baseline	4359.3 ± 397.9	4464.7 ± 469.9	4215.2 ± 681.4	4127.3 ± 579.7
11 w	4265.1 ± 438.2	3561.5 ± 440.6*	3621.5 ± 582.4*	3655.7 ± 492.8*
12 w	4366.7 ± 418.3	3389.9 ± 380.9*	3878.4 ± 526.2* ^#^	3335.7 ± 499.0* ^##^
LVESV (ml)				
baseline	0.25 ± 0.06	0.25 ± 0.08	0.25 ± 0.08	0.24 ± 0.08
11 w	0.25 ± 0.05	0.36 ± 0.09*	0.35 ± 0.05*	0.35 ± 0.07*
12 w	0.26 ± 0.06	0.38 ± 0.08*	0.31 ± 0.07* ^#^	0.37 ± 0.07* ^##^
LVEDV (ml)				
baseline	1.08 ± 0.07	1.08 ± 0.08	1.07 ± 0.07	1.09 ± 0.07
11 w	1.09 ± 0.08	1.21 ± 0.06*	1.20 ± 0.09*	1.19 ± 0.06*
12 w	1.09 ± 0.07	1.20 ± 0.08*	1.14 ± 0.06* ^#^	1.17 ± 0.08*
LVEF (%)				
baseline	76.5 ± 5.8	76.8 ± 7.3	76.0 ± 7.5	78.1 ± 7.1
11 w	75.3 ± 5.2	68.2 ± 7.5*	67.4 ± 7.8*	67.1 ± 6.4*
12 w	75.5 ± 4.9	67.9 ± 6.0*	72.1 ± 5.7^#^	68.5 ± 5.4* ^##^

LVESV, left ventricular end-systolic volume; LVEDV, left ventricular end-diastolic volume; LVEF, left ventricular ejection fraction; Values are presented as mean ± SD. *p < 0.05 *vs *Control, ^# ^*p *< 0.05 *vs *DM, ^## ^*p *< 0.05 *vs *TSN.

LVEF, ESV, EDV were evaluated by echocardiography 12 w after STZ injection. Table 2 shows that TSN significantly improved LVEF in diabetic rats compared with the diabetes group 12 w after STZ injection, with LVEF reaching levels similar to those of the control animals (72.1 ± 5.7 *vs *75.5 ± 4.9, *P *> 0.05). HOE140 abrogated the effects of TSN on LVEF enhancement (68.5 ± 5.4 in HOE140 group *vs *67.9 ± 6.0 in diabetes group, *P *> 0.05). TSN significantly inhibited the increase of LVESV compared with the diabetes group (0.31 ± 0.07 *vs *0.38 ± 0.08 ml, *P *< 0.05) and the HOE140 group (0.31 ± 0.07 *vs *0.37 ± 0.07 ml, *P *< 0.05). Larger volume of LVEDV were observed in the diabetes group (1.20 ± 0.08 *vs *1.14 ± 0.06 ml, *P *< 0.05) and the HOE140 group (1.17 ± 0.08 *vs *1.14 ± 0.06 ml, *P *> 0.05) as compared with in the TSN group.

### Antiapoptotic effect of TSN on cardiomyocytes in rats with diabetes

**Figure 2:**
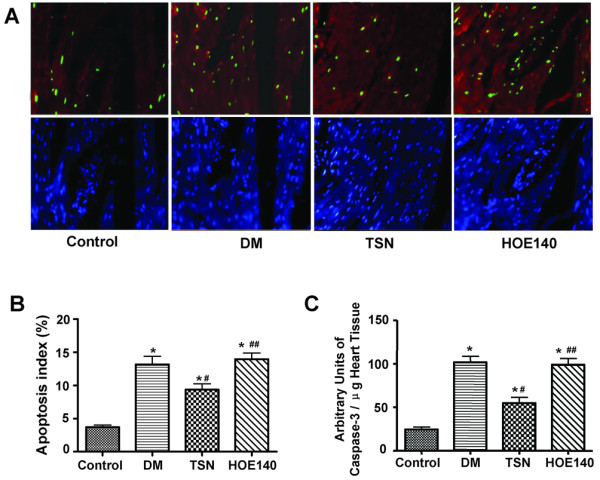
**Antiapoptotic effect of TSN on cardiomyocytes in diabetic rats**. TSN reduced the number of apoptotic cardiomyocytes compared with the diabetes group and the HOE140 group (A). The number of TUNEL-positive cardiomyocytes (in green, DAPI in blue) was significantly less in the TSN group than in the diabetes group and the HOE140 group (B). TSN treatment significantly decreased caspase-3 activity compared with the diabetes group and the HOE140 group (C). The columns and errors bars represent means and SD. *p < 0.05 *vs *control, ^# ^*p *< 0.05 *vs *diabetes, ^## ^*p *< 0.05 *vs *TSN.

Representative results are shown in Figure 2A. STZ-induced diabetes resulted in markedly increased TUNEL-positive myocytes compared with the TSN group (0.132 ± 0.039 *vs *0.094 ± 0.028, *P *< 0.05) (Figure 2B) demonstrated by quantitative analyses. Co-administration of HOE140, significantly abolished the antiapoptotic effect of TSN (0.139 ± 0.030 in HOE140 group *vs *0.094 ± 0.028 in TSN group, *P *< 0.05) (Figure 2B). Concurrently, levels of caspase-3 activity were determined and we found that TSN group showed significantly decreased caspase-3 enzymatic activity compared with the diabetes group (55.0 ± 20.7 *vs *101.7 ± 22.0, *P *< 0.05) and the HOE140 group (55.0 ± 20.7 *vs *98.9 ± 22.3, *P *< 0.05) (Figure 2C).

### TSN protected the cardiomyocytes ultrastructure against the damage induced by diabetes

**Figure 3:**
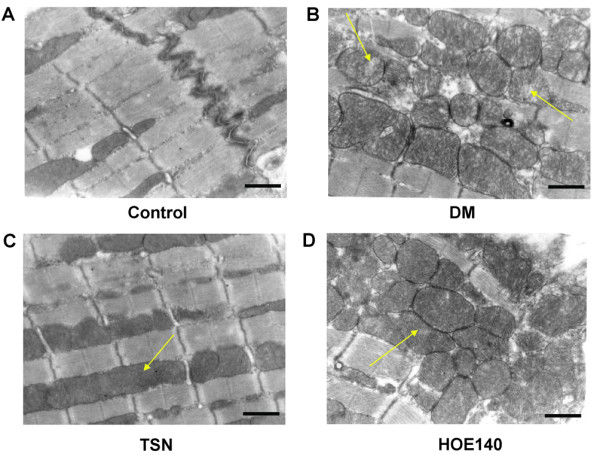
**TSN protected the cardiomyocytes ultrastructure against the damage induced by diabetes**. Electron micrographs of control hearts (A) showed regular myofibrillar organization with evident Z-lines (white arrow) and normal mitochontria profiles (yellow arrow). In STZ-diabetic hearts (B) and HOE140 pretreatment hearts (D), great size mitochondria with the destruction of cristae were detectable. The ultrastructural appearance of the TSN-treated hearts were similar to controls (C), indicating that the protective effect on the myocardium preserves the ultrastructure of cardiomyocytes. Scale bars, 0.5 μm.

Electron microscope showed that the number of cardiomyocyte mitochondria was increased in the diabetic group. Cardiomyocyte mitochondria were found greater in size with the destruction of cristae in experimental diabetic cardiomyopathy (Figure 3A, B). While TSN pretreatment alleviated mitochondria ultrastructure changes with intact membrane and cristae although still with a greater size which could be reversed by HOE140 (Figure 3C, D).

### TSN treatment increases Akt and GSK-3β phosphorylation, decreases NF-κB phosphorylation, reduces cytokine levels and alleviates leukocyte infiltration after I/R injury in diabetic rats

**Figure 4:**
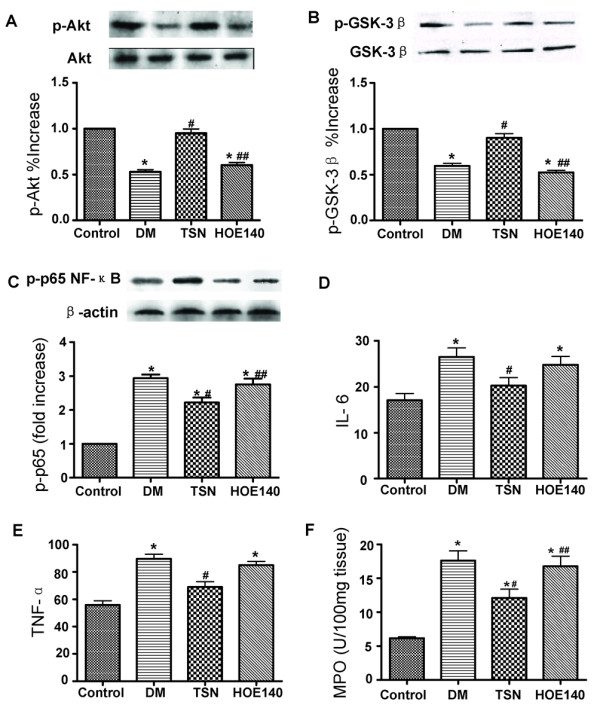
**Leukocyte infiltration and inflammation cytokines were alleviated after TSN Administration**. A significant increase in phosphorylation of Akt (Ser 473) (A) and GSK-3β (B) were observed in myocardial tissue from hearts pretreated with TSN. Administration of HOE140 shortly before TSN pretreatment abolished the effects of TSN. The levels of phospho-NF-κB p65 protein were elevated in the diabetes group compared with the control group. TSN administration also associated with reduced expression of phospho-NF-κB p65 protein and this effect was abolished by HOE140 administration. (C). Consistent with the changes of NF-κB p65 phosphorylation, diabetes resulted in a noticeable increase in IL-6, TNF-α and MPO compared with control group. TSN significantly reduced the levels of IL-6, TNF-α and MPO compared with the diabetes group. However, HOE140 administration significantly increasd IL-6, TNF-α and MPO level compared with the TSN group (D, E, F). The columns and errors bars represent means and SD. *p < 0.05 *vs *control, ^# ^*p *< 0.05 *vs *diabetes, ^## ^*p *< 0.05 *vs *TSN.

To further elucidate the potential mechanism of cardio-protective effects of TSN. We investigated the effects of TSN on the kinin B2 receptor-Akt-GSK-3β signalling pathway. TSN treatment was associated with a significant increase in phosphorylation of Akt in cardiac tissue compared with the diabetes group as determined by western blot analysis. However, with the addition of HOE140, p-Akt expression was significantly lower than when the pretreatment was carried out with TSN alone (Figure 4A). The levels of p-GSK-3β protein were elevated in the TSN group which indicated GSK-3β activation. The effect of TSN on p-GSK-3β was inhibited by HOE140 administration (Figure 4B). TSN administration also associated with reduced expression of phospho-NF-κB p65 protein and this effect was abolished by HOE140 administration as indicated in Figure 4C. ELISA was employed to measure the levels of IL-6 and tumor necrosis factor-α (TNF-α) (Figure 4D, E). Diabetes resulted in a noticeable increase in IL-6 (26.5 ± 6.3 vs 17.1 ± 4.7 pg/mg protein, *P *< 0.05) and TNF-α (89.7 ± 10.2 vs 55.9 ± 9.5 pg/mg protein, *P *< 0.05) compared with the control group. TSN decreased the levels of IL-6 (20.3 ± 5.5 vs 26.5 ± 6.3 pg/mg protein, *P *< 0.05) and TNF-α (69.0 ± 12.4 vs 89.7 ± 10.2 pg/mg protein, *P *< 0.05) compared with the diabetes group. HOE140 abolished the effect of TSN, a increased production of IL-6 (24.8 ± 5.7 vs 20.3 ± 5.5 pg/mg protein, *P *< 0.05) and TNF-α (84.9 ± 9.1 vs 69.0 ± 12.4 pg/mg protein, *P *< 0.05) as compared with the TSN group. The activity of MPO was significantly elevated in the diabetes group when compared to the control group (17.6 ± 4.6 *vs *6.2 ± 0.6 U/100 mg, *P *< 0.05). TSN pretreatment reduced MPO activity as compared with the diabetes group (12.1 ± 4.1 *vs *17.6 ± 4.6 U/100 mg, *P *< 0.05). Co-administration with HOE140 abrogated the effects of TSN (16.8 ± 4.8 *vs *12.1 ± 4.1 U/100 mg, *P *< 0.05) (Figure 4F).

## Discussion

Cardiovascular complications remains the leading cause of diabetes-related mortality and morbidity [15]. The belief is widely held that the increase in cardiovascular mortality is a consequence of accelerated atherosclerosis. However, a specific disease termed as diabetic cardiomyopathy, increases the risk for cardiac dysfunction and heart failure independently of other risk factors such as coronary artery disease and hypertension as evidenced by compelling epidemiological and clinical data [16,17]. Despite the potential importance of this disease entity, the underlying mechanisms are still not well understood. Disruption of the extracellular matrix regulation with accumulation of cardiac collagen, and furthermore cardiac inflammation may be an important mediator of this disease.

Salvia miltiorrhiza (Danshen) is an annual sage plant which grows in China, Mongolia, Korea and Japan. Chemical constituents from S. miltiorrhiza root extract are classified into 2 major categories: water-soluble compounds (WSC) and lipophilic diterpenoid quinines (LDQ), the compounds of both have been mostly identified and purified [18]. Among the major diterpenes isolated, including cryptotanshinone, tanshinone I, tanshinone IIA and dihydrotanshinone, tanshinone IIA had been shown to posses various pharmacological activities.

Our previous work has shown that TSN pretreatment reduces infarct size and improves cardiac dysfunction after I/R injury in diabetic rats. This was accompanied by decreased cardiac apoptosis and inflammation. The possible mechanism responsible for the effects of TSN is associated with the phosphatidylinositol 3-kinase (PI3K)/Akt/NF-κB-dependent pathway[19]. In addition, TSN protects against cardiotoxicity induced by doxorubicin in vitro and in vivo concluded by Jiang and colleagues [20]. Hong [21] et al. reported that tanshinone IIA prevents doxorubicin induced cardiomyocyte apoptosis through Akt-dependent pathway. Moreover, TSN also has anticancer properties evidenced by inhibiting the proliferation of mouse P388 lymphocytic leukemia cells [22], and inducing apoptosis of human hepatocellular carcinoma cells [23], etc. Although cardiomyocyte apoptosis and inflammatory reaction were increased in experimental model of diabetic cardiomyopathy and TSN has anti-apoptosis and anti-inflammation properties. The effects of TSN on experimental diabetic cardiomyopathy and the exact mechanism of its therapeutic action are still poorly understood. This promoted an investigation of the protective effects of TSN on experimental diabetic cardiomyopathy and the underlying mechanism.

In the present study, TSN attenuates cardiac systolic and diastolic dysfunction in experimental diabetic cardiomyopathy. TSN treated rats had significantly smaller LVEDV and LVESV increases and LVEF decrease 12 w after STZ injection versus diabetic rats. The decrease in + LV dp/dt max and - LV dp/dt max also tended to be smaller in TSN treated rats as compared with diabetic rats, showing a protective effect of TSN on cardiac function. Evidence from experimental models and human cardiac disease shows that cardiomyocytes' loss as a result of apoptosis is significant in various heart diseases and inevitably leads to heart failure [24,25]. Blocking the apoptosis process could prevent the loss of contractile cells, minimize cardiac injury and therefore slow down or even prevent the occurrence of heart failure [26,27]. Thus, we performed TUNEL staining and measured caspase 3 activity in order to explore the underlying mechanism responsible for the cardiac function improvement induced by TSN in diabetic rats. The results indicated that TSN decreased cardiomyocyte apoptotic index in diabetic rats which was in agreement with our previous work which showing potent cardio-protective effects of TSN. TSN pretreatment alleviated mitochondria ultrastructure changes caused by diabetes as well.

In the present study, diabetic cardiomyopathy is characterized by an decrease in the phosphorylation state of Akt and GSK-3β, which was associated with augmented cardiac inflammation as evidenced by increased NF-κB phosphorylation and TNF-α, IL-6 expression, as well as increased MPO activity. These changes were normalized by TSN administration. TSN enhanced Akt and GSK-3β phosphorylation, inhibited NF-κB phosphorylation and decreased TNF-α, IL-6 expression and inhibited MPO activity.

Diabetes mellitus is a growing public health problem that needs to be tackled at multiple levels such as prevention and health maintenance and aggressive management of associated comorbidities [16,17,28]. The kallikrein-kinin system (KKS), is known to attenuate, e.g., cardiac inflammation, fibrosis, apoptosis, and hypertrophy when the system is artificially intensified [29]. The biological effects of kinins in man are mediated by two G protein coupled receptors, B1and B2. Among these two receptors, B2 receptor in constitutively expressed on the surface of many cell types under physiological conditions [30]. Evidence shows that the B2R is beneficial in myocardial diseases, protecting from inflammation, fibrosis and apoptosis, while B1R shows a proinflammatory character contributing to the disease progression by increasing the production of cytokines and stimulating the migration of immune cells [31]. HOE140, a specific kinin B2 receptor antagonist, blocks the vasodilatation and increased vascular permeability associated with exogenous bradykinin administration both in experimental models and *in vivo *in man [32].

In the present study, HOE140 abolished the beneficial effects of TSN: a decrease in LVEF and ± dp/dt, an inhibition of cardiomyocyte apoptosis, a destruction of cardiomyocyte mitochondria cristae, a reduction of Akt and GSK-3β phosphorylation, an enhancement of NF-κB phosphorylation and an increase of TNF-α, IL-6 and MPO production. Therefore, the kinin B2 receptor could constitute potential therapeutic targets in the treatment of diabetic cardiomyopathy. This information provides important insights regarding the role and mechanism of TSN in protection against diabetic cardiomyopathy.

## Limitation

The present study only focused on the kinin B2 receptor-Akt-GSK-3β signaling pathway. Some other pathways may also participated in the pathogenesis of diabetic cardiomyopathy such as MAPK related pathway, etc [33]. Further studies should focus on different pathways to elucidate more treatment targets of diabetic cardiomyopathy. Right ventricular function is an important factor in evaluating the prognosis of cardiomyopathy [34]. Thus, right ventricular function should be assessed in the following studies to systematically evaluate the heart function of diabetic cardiomyopathy. Moreover, the key factors inactivated Akt/GSK-3beta/NF-κB involving increased IL-6, TNF-αand MPO still need to be clarified in the future studies.

## Conclusions

Our findings underscore the cardioprotective effects of TSN. TSN improves cardiac performance by inhibiting apoptosis, alleviating mitochondria ultrastructure changes and reducing inflammatory cytokine production in experimental diabetic cardiomyopathy. TSN induced cardio-protective effects are mediated, at least in part, through the kinin B2 receptor-Akt-GSK-3β signalling pathway.

## Abbreviations Used

AI: apoptotic index; Akt: protein kinase B; B1R: kinin B1 receptor; B2R: kinin B2 receptor; Danshen: salvia miltiorrhiza; DM: diabetes mellitus; ± dp/dt: maximum speed of contraction/relaxation; GSK-3β: glycogen synthase kinase 3β; IL-6: interleukin 6; I/R: ischemia reperfusion; KKS: kallikrein-kinin system; LDQ: lipophilic diterpenoid quinines; LVEDD: left ventricular end-diastolic diameter; LVEDP: left ventricular end-diastolic pressure; LVEDV: left ventricular end-diastolic volume; LVEF: left ventricular ejection fraction; LVESD: left ventricular end-systolic diameter; LVESV: left ventricular end-systolic volume; LVP: left ventricular pressure; LVSP: left ventricular systolic pressure; MPO: myeloperoxidase; NF-κB: nuclear factor kappa-light-chain-enhancer of activated B cells; PI3K: phosphatidylinositol 3-kinase; SD: sprague-dawley; STZ: streptozocin; TNF-α: tumor necrosis factor-α; TSN: tanshinone IIA; TUNEL: terminal deoxyribonucleotidyl transferase-mediated dUTP-biotin nick end labeling; WSC: water-soluble compounds.

## Competing interests

The authors declare that they have no competing interests.

## Authors' contributions

DDS participated in the design of the study, statistical analysis, drafted the manuscript and performed electron microscopy study. MS, JYL carried out the apoptosis and cardiac function studies. YMZ performed western blot analysis. JL, ZZ, LZ, YY performed the animal handling, drug administration and ELISA Detection. HCW, FC participated in its design and coordination and helped to draft the manuscript. All authors read and approved the final manuscript.
